# Two-point fixed pulley-traction method in endoscopic submucosal dissection for early gastric neoplasm

**DOI:** 10.1055/a-2173-8010

**Published:** 2023-09-27

**Authors:** Tsubasa Takeuchi, Mitsuru Esaki, Yosuke Minoda, Yoshitaka Hata, Haruei Ogino, Eikichi Ihara, Yoshihiro Ogawa

**Affiliations:** 1 Department of Medicine and Bioregulatory Science, Graduate School of Medical Sciences, Kyushu University, Fukuoka, Japan; 2 Department of Gastroenterology, Harasanshin Hospital, Fukuoka, Japan; 3 Department of Gastroenterology and Metabolism, Graduate School of Medical Sciences, Kyushu University, Fukuoka, Japan

Endoscopic submucosal dissection (ESD) is a technically challenging procedure with substantial risk of intraoperative complications. Traction assistance is a promising strategy for simplifying ESD. Although multiple traction methods have been proposed 1 2 3 4 5 , there is room for further optimization. We designed a novel traction method termed the two-point fixed pulley-traction method (TPPT), which is detailed below and illustrated in Video 1 .

**Video 1** Two-point fixed pulley-traction method in endoscopic submucosal dissection for early gastric neoplasm.


We implemented TPPT during ESD in an 80-year-old man with an early gastric neoplasm. The lesion measured 10 mm and was located on the lesser curvature of the gastric body. A viscous solution was injected into the submucosal layer and a circumferential mucosal incision was made around the lesion ( Fig. 1 a ). TPPT was implemented as follows. First, a clip hooking a small base ring of silicon bands was placed on the mucosal flap at one side of the lesion ( Fig. 1 b ). A thread had been pre-tied to the central ring of the silicon bands ( Fig. 2 ). Second, another clip, hooking the distal ring, was placed on the opposite side of the mucosal flap ( Fig. 1 c ). Finally, a third clip hooking the thread was placed on the greater curvature, opposite the lesser curvature bearing the lesion. TPPT was finalized by pulling the thread ( Fig. 1 d ). TPPT applied a stable vertical traction force to the target lesion, enhancing the visibility of the submucosal layer until the completion of submucosal dissection. Consequently, en bloc resection was performed efficiently without complications.

**Fig. 1:**
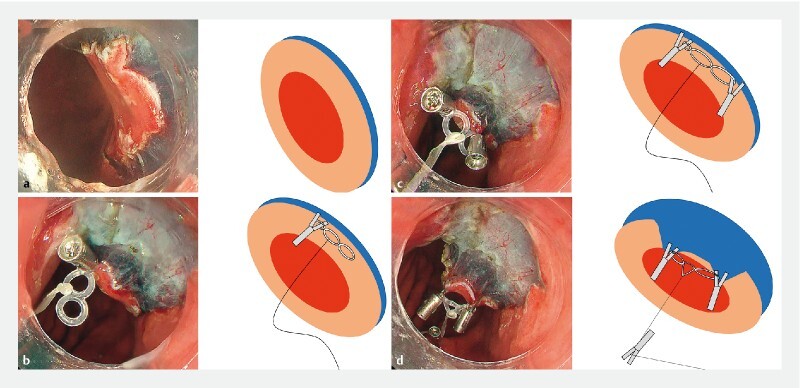
Steps of the two-point fixed pulley-traction method. **a** A circumferential mucosal incision was made around the lesion. **b** First clip hooking the base: a small ring of silicon bands, with a thread pre-tied to the central ring, was placed on one side of the mucosal flap. **c** A second clip hooking the distal ring was placed on the other side of the mucosal flap. **d** The third clip hooked the thread and was placed on the greater curvature of the stomach, opposite the lesser curvature bearing the lesion.

**Fig. 2:**
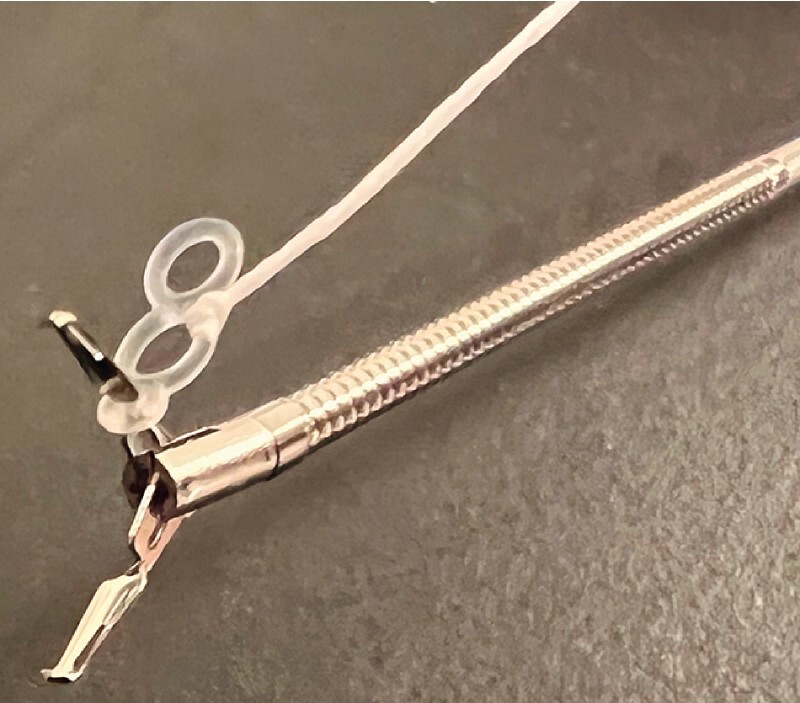
Clip with traction band and pre-tied thread.

TPPT is a traction method that combines a two-point traction strategy with a pulley system. The entire mucosal flap could be elevated by applying traction at two points. Additionally, a vertical traction force was obtained via the pulley system, offering optimal traction. Therefore, TPPT could potentially serve as a useful tool for assisting ESD procedures for early gastric neoplasms.

Endoscopy_UCTN_Code_TTT_1AO_2AG

## Notes

Endoscopy E-Videos

https://eref.thieme.de/e-videos
*E-Videos* is an open access online section of the journal *Endoscopy* , reporting on interesting cases and new techniques in gastroenterological endoscopy. All papers include a high-quality video and are published with a Creative Commons CC-BY license. Endoscopy E-Videos qualify for HINARI discounts and waivers and eligibility is automatically checked during the submission process. We grant 100% waivers to articles whose corresponding authors are based in Group A countries and 50% waivers to those who are based in Group B countries as classified by Research4Life (see: https://www.research4life.org/access/eligibility/ ). This section has its own submission website at https://mc.manuscriptcentral.com/e-videos
